# Food Group, Macronutrient Intake, and Metabolic Status in the US-Affiliated Pacific's Children's Healthy Living (CHL) Program

**DOI:** 10.1093/jn/nxac173

**Published:** 2022-08-04

**Authors:** Rachel Novotny, Ashley B Yamanaka, Rica Dela Cruz, Sabine Strasburger, Carol J Boushey, Jean Butel, Monica Esquivel, Tanisha F Aflague, Travis Fleming, Patricia Coleman, Jonathan Deenik, Leslie Shallcross, Lynne R Wilkens

**Affiliations:** Human Nutrition, Food and Animal Sciences Department, University of Hawaii at Manoa, Honolulu HI, Hawai ʻi; Human Nutrition, Food and Animal Sciences Department, University of Hawaii at Manoa, Honolulu HI, Hawai ʻi; Human Nutrition, Food and Animal Sciences Department, University of Hawaii at Manoa, Honolulu HI, Hawai ʻi; Human Nutrition, Food and Animal Sciences Department, University of Hawaii at Manoa, Honolulu HI, Hawai ʻi; University of Hawai‘I Cancer Center; Human Nutrition, Food and Animal Sciences Department, University of Hawaii at Manoa, Honolulu HI, Hawai ʻi; Human Nutrition, Food and Animal Sciences Department, University of Hawaii at Manoa, Honolulu HI, Hawai ʻi; University of Guam; American Samoa Community College; Northern Marianas College; Human Nutrition, Food and Animal Sciences Department, University of Hawaii at Manoa, Honolulu HI, Hawai ʻi; University of Alaska at Fairbanks; University of Hawai‘I Cancer Center

**Keywords:** nutrient, food group, children, Pacific, obesity

## Abstract

**Background:**

The Children's Healthy Living study provided dietary intake information for understudied Native Hawaiian and Other Pacific Islander (NHOPI) young children.

**Objectives:**

Our objective was to describe food group and macronutrient intakes of NHOPI children in the US-Affiliated Pacific region (USAP), overall and by jurisdiction, income level, and metabolic status.

**Methods:**

We evaluated 2–8-y-olds (*n* = 3520) in a cross-sectional cluster sampled study using 2 d of dietary records completed by caregivers using provided tools, quantified by a specially developed food composition table and compared with US dietary recommendations. Overweight and obesity (OWOB) and acanthosis nigricans (AN) assessment (metabolic status) was completed by trained evaluators using standard tools. Demographic data were collected by questionnaire. Regression analysis identified differences in dietary component means by jurisdiction, World Bank income groups (WBIGs), and metabolic status, adjusted for age and sex.

**Results:**

Few children met US recommendations for vegetable (2.4%) and milk (4.1%) food groups. US macronutrient recommendations were generally met. Food group and macronutrient intakes were significantly different by jurisdiction and WBIG. Means for food groups, except meats, and macronutrients, except protein, were higher in overweight/obese (OWOB) compared with healthy-weight children. Grain intake of 7.25 (SE: 0.08) oz was higher (*P* < 0.05) and milk intake of 0.90 (SE: 0.05) cups was lower (*P* < 0.05) in children with OWOB compared with those without OWOB [grains: 7.17 (SE: 0.07) oz; and milk: 0.96 (SE: 0.04) cups]. Monounsaturated fat intake of 11.68 (SE: 0.10) % energy was higher in those with OWOB, compared with healthy-weight children [11.56 (SE: 0.08) % energy, *P* < 0.05].

**Conclusions:**

Young children's diets in the USAP did not meet milk, vegetable, or fruit intake recommendations. There was variability in dietary patterns across the USAP and by WBIG. Grain intake and monounsaturated fat intake were lower and milk intake was higher in children with better metabolic status.

## Introduction

The US federal government last collected dietary data on children in Hawai‘i and Alaska in the Nationwide Food Consumption Survey of 1977–78. These data were never collected in the wider US-Affiliated Pacific region (USAP) (1), which includes the Pacific island US territories of Guam, American Samoa, and the Commonwealth of the Northern Mariana Islands (CNMI); and the Pacific island nations of the Republic of Palau, Republic of the Marshall Islands (RMI), and Federated States of Micronesia (Chuuk, Kosrae, Pohnpei, Yap). The latter nations are in a compact of free association with the United States and are considered Freely Associated States (FAS) (2). The region ranges from lower-middle-income (LMI) to high-income (HI) status according to the World Bank income categories (3).

The lack of data collection has resulted in no federal record of dietary intakes of children in the region (4), even for the states of Hawai‘i and Alaska, in the last 50 y. Clearly the food environment has changed dramatically, with evidence of nutrition transition (5–7), as has the prevalence of obesity and acanthosis nigricans (AN) (an indicator of insulin resistance), with considerable variability across jurisdictions and World Bank income levels (8). Worldwide trends have shown that the prevalence of child obesity is higher in HI and upper-middle-income (UMI) countries compared with LMI countries. In contrast, stunting is more prevalent in LMI countries than UMI or HI countries (9).

A few studies in 2002–2008 collected dietary intake and assessed nutrient intakes of children in the USAP region. Micro- or macronutrient assessments of children's diets were done in Hawai‘i (10–12), Guam (13), CNMI (14), and RMI (15), with sample sizes ranging from 116 to 954. Most of the research on child nutrition in the USAP was conducted in Hawai‘i (10–12) or focused on children of adolescent age (10, 11, 13). Only 1 nutrient intake study focused on young children aged 6 mo to 10 y (14). Another study included an environmental intervention targeting food stores and assessed the change in nutrient intake of children (12). The majority of the USAP jurisdictions, including Alaska, American Samoa, and most of the FAS, have no published studies on child nutrient intake. Most studies related to nutrition in the USAP region have focused on weight outcomes such as obesity and have not provided detailed nutrient profiles.

The Children's Healthy Living (CHL) Program fills a gap in food and nutrient intake information for young children in this region. These data are vital for program and policy planning. Documentation of dietary intake and nutrient profiles of children in the USAP are needed to better understand the nutrition transition and weight trends occurring within the USAP region and to guide nutrition programs. The purpose of this article is to describe food group and macronutrient intakes in young children participating in CHL in the USAP, overall and by jurisdiction, World Bank income group, and metabolic status.

## Methods

### Study population

Cross-sectional data were collected on 2–8-y-old children recruited from 33 communities in 11 USAP jurisdictions (Hawai‘i, Alaska, CNMI, Guam, American Samoa, Palau, RMI, and the 4 Federated States of Micronesia: Pohnpei, Yap, Kosrae, and Chuuk) in a community cluster design. Twenty-seven communities were selected in 2011 from the 5 US state and territory jurisdictions to participate in the CHL randomized controlled trial (16). Communities were selected for the CHL Trial in Guam, Hawai‘i, Alaska, American Samoa, and in CNMI. Selection was based on 2000 US census data for population sizes >1000, relative accessibility and representativeness, having >25% of the population of indigenous/native descent (15% in Alaska due to focus on accessible communities), and having >10% of the population aged <10 y. The minimum sample size of 150 children per community for anthropometry and 100 children per community for diet was selected to provide sufficient power for the CHL Trial (16, 17). Generally, the first 100 children provided dietary data. The sample size was set in order to detect important differences between test and control communities in the community randomized intervention, with α = 0.05 (2-sided) and β = 0.20. For an Intraclass Correlation (ICC) within community of 0.02 based on prior studies, 100 children per community leads to a modest effect size of 0.31, which reflects a difference of 0.5 servings of vegetables, and 150 children per community, of 0.26, which reflects a difference of 0.09 for BMI *z*-score.

An additional prevalence assessment (Prevalence Study) in 2013 was intended to provide representative samples from the 6 additional USAP jurisdictions, to participate in the same data collection protocol and methods as the baseline of the CHL Trial (16, 17). To achieve geographic representation, the main population centers/islands were divided into sectors for sampling, with the number of children recruited in each sector proportional to the number of children in the sector based on 2010 country-specific censuses. For the jurisdictions of Yap and RMI, a remote island was additionally sampled. Each FAS jurisdiction has a majority indigenous population and a relatively young population, and therefore meets the CHL criteria of >25% of the population of indigenous/native descent and >10% of the population under age 10 y. Availability of scheduled air or boat service was an additional selection criterion for sampling sectors in the FAS. A total sample size of 200 per jurisdiction was selected to provide reasonably precise prevalence estimates.

**FIGURE 1:**
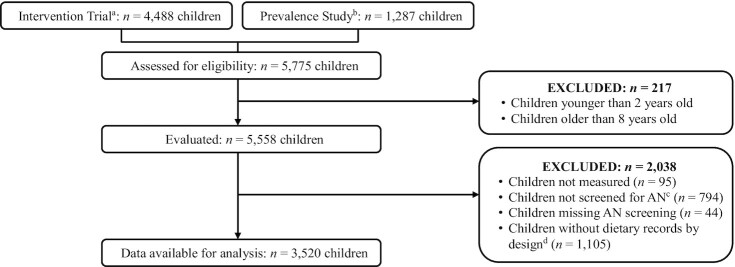
Flowchart of study selection (*n* = 3520) from the Children's Healthy Living Program. ^a^Baseline data. Jurisdictions: Alaska, American Samoa, CNMI, Guam, and Hawai‘i. ^b^Jurisdictions: Chuuk, Kosrae, Pohnpei, Republic of Palau, Republic of the Marshall Islands, Yap. ^c^Some communities in the intervention trial were only assessed for demographic information and anthropometry. ^d^100 children of ∼150 per community were assessed for diet. AN, acanthosis nigricans; CNMI, Commonwealth of the Northern Mariana Islands.

A total of 5775 children participated from the 33 communities; 4488 children were from the jurisdictions participating in the CHL Trial, from which baseline data were used (**Figure 1**) (16). The other 1287 children were from jurisdictions participating in the CHL Prevalence Study.

Of the 5775 children, 217 children were excluded because their age as reported by the caregiver was outside the study age range. Of the remaining 5558, there were 5463 children (51.1% male) measured for anthropometry and 4625 (50.8% male) screened for AN. The AN number is smaller by design because some communities in the Intervention Study were only measured for anthropometry. Dietary records were obtained on a subsample of 3520 children from the 11 jurisdictions and are used in this study (**Table 1**).

**TABLE 1:** Description of the study sample by Pacific jurisdiction and World Bank income groups^1^

Characteristic	Lower-middle-income jurisdictions				Upper-middle-income jurisdictions			High-income jurisdictions				Total
	Chuuk	Kosrae	Pohnpei	Yap	American Samoa	Palau	RMI	Alaska	CNMI	Guam	Hawai‘i	
Total, *n*	123	125	187	190	588	166	191	308	546	666	430	3520
Sex, *n* (%)												
Male	70 (56.9)	71 (56.8)	91 (48.7)	88 (46.3)	306 (52)	89 (53.6)	82 (42.9)	173 (56.2)	283 (51.8)	339 (50.9)	203 (47.2)	1795 (51)
Female	53 (43.1)	54 (43.2)	96 (51.3)	102 (53.7)	282 (48)	77 (46.4)	109 (57.1)	135 (43.8)	263 (48.2)	327 (49.1)	227 (52.8)	1725 (49)
Age group, *n* (%)												
2–5 y	70 (56.9)	78 (62.4)	167 (89.3)	135 (71.1)	341 (58)	124 (74.7)	69 (36.1)	228 (74)	336 (61.5)	348 (52.3)	297 (69.1)	2193 (62.3)
6–8 y	53 (43.1)	47 (37.6)	20 (10.7)	55 (29)	247 (42)	42 (25.3)	122 (63.9)	80 (26)	210 (38.5)	318 (47.8)	133 (30.9)	1327 (37.7)
Indigenous,^2^ *n* (%)												
Yes	121 (98.4)	122 (97.6)	183 (97.9)	186 (97.9)	579 (98.5)	157 (94.6)	191 (100)	68 (22.1)	320 (58.6)	444 (66.7)	320 (74.4)	2691 (76.5)
No	2 (1.6)	3 (2.4)	4 (2.1)	4 (2.1)	9 (1.5)	9 (5.4)	0	240 (77.9)	226 (41.4)	222 (33.3)	110 (25.6)	829 (23.6)
Income per year, *n* (%)												
<$35,000	37 (30.1)	96 (76.8)	121 (64.7)	108 (56.8)	450 (76.5)	149 (89.8)	98 (51.3)	151 (49.0)	389 (71.3)	334 (50.2)	215 (50.0)	2148 (61.0)
≥$35,000	2 (1.6)	0	8 (4.3)	0	27 (4.6)	8 (4.8)	7 (3.7)	136 (44.2)	36 (6.6)	134 (20.1)	172 (40.0)	530 (15.1)
Missing	84 (68.3)	29 (23.2)	58 (31.0)	82 (43.2)	111 (18.9)	9 (5.4)	86 (45.0)	21 (6.8)	121 (22.2)	198 (29.7)	43 (10.0)	842 (23.9)
BMI category (40), *n* (%)												
Underweight	5 (4.1)	5 (4)	0	12 (6.3)	2 (0.3)	4 (2.4)	14 (7.3)	2 (0.7)	24 (4.4)	19 (2.9)	12 (2.8)	99 (2.8)
Healthy weight	107 (87)	106 (84.8)	139 (74.3)	152 (80)	330 (56.1)	121 (72.9)	171 (89.5)	200 (64.9)	380 (69.6)	463 (69.5)	280 (65.1)	2449 (69.6)
Overweight	9 (7.3)	8 (6.4)	30 (16)	12 (6.3)	105 (17.9)	11 (6.6)	5 (2.6)	60 (19.5)	54 (9.9)	84 (12.6)	71 (16.5)	449 (12.8)
Obese	1 (0.8)	6 (4.8)	7 (3.7)	8 (4.2)	149 (25.3)	26 (15.7)	0	38 (12.3)	87 (15.9)	87 (13.1)	65 (15.1)	474 (13.5)
Missing	1 (0.8)	0	11 (5.9)	6 (3.2)	2 (0.3)	4 (2.4)	1 (0.5)	8 (2.6)	1 (0.2)	13 (2)	2 (0.5)	49 (1.4)
Acanthosis nigricans present, *n* (%)												
Yes	4 (3.3)	4 (3.2)	22 (11.8)	4 (2.1)	47 (8)	11 (6.6)	22 (11.5)	0	51 (9.3)	21 (3.2)	6 (1.4)	192 (5.5)
No	118 (95.9)	121 (96.8)	160 (85.6)	180 (94.7)	540 (91.8)	152 (91.6)	168 (88)	305 (99)	495 (90.7)	640 (96.1)	420 (97.7)	3299 (93.7)
Missing	1 (0.8)	0	5 (2.7)	6 (3.2)	1 (0.2)	3 (1.8)	1 (0.5)	3 (1)	0	5 (0.8)	4 (0.9)	29 (0.8)

1 CNMI, Commonwealth of the Northern Mariana Islands; RMI, Republic of the Marshall Islands.

2 Indigenous: culturally distinctive ethnicities that originated from that jurisdiction.

### Race/ethnicity

Caregivers reported the race/ethnicity of children by completing a questionnaire that provided checkboxes according to the Office of Management and Budget categories (16). Additional subcategories of common ethnic groups of the region, as well as a write-in space, were provided under Asian, Native Hawaiian and Other Pacific Islanders, and American Indian/Alaska Native. An indigenous variable was developed from the Pacific Islander and Alaskan Native ethnicities, indicating the ethnicity matching the jurisdiction in which the data were collected (Palau: Palauan; Yap: Yapese; GU: Guam; CHamoru/Chamorro; CNMI: CHamoru/Chamorro and Carolinian; Chuuk: Chuukese; Pohnpei: Pohnpeian; Kosrae: Kosraean; RMI: Marshallese; American Samoa: Samoan; Hawai‘i: Native Hawaiians; Alaska: American Indian or Alaskan Native).

### World Bank income groups

The UN World Bank classifies country economies into 4 categories—World Bank income groups (WBIGs)—based on estimates of gross national income per capita (GNIPC) for 2015. Low-income economies were those with a GNIPC ≤$1045; LMI economies were those with a GNIPC between $1046 and $4125; UMI economies were those with a GNIPC between $4126 and $12,735; and HI economies were those with a GNIPC ≥$12,736 (3). LMI jurisdictions participating in CHL included all states of the Federated States of Micronesia (Yap, Chuuk, Pohnpei, and Kosrae). UMI jurisdictions included American Samoa, the RMI, and Palau. HI jurisdictions included Guam, CNMI, Hawai‘i, and Alaska (3, 18). Because jurisdiction income and household income were highly correlated, only jurisdiction income is used in this article.

### Anthropometry

Weight (kilograms) and height (centimeters) were measured according to standard procedures (22) and equipment (Perspective Enterprises Stadiometer model PE-AIM-101, SECA scale Model 876) throughout the USAP region. All anthropometric measurers were standardized to 1 expert with intrameasurer reliability of 0.999 for both weight and height, and measurement equipment was calibrated prior to each measurement session (19). Three measurements each for height and weight were taken; if 2 of the 3 were not within 0.2 units (20 mm or 200 g, respectively), the first 3 measurements were crossed out and an additional set of 3 measurements was taken. All measurements were averaged for analysis. The BMI was calculated as kg/m^2^. Categories were defined using CDC control reference BMI percentile data according to age and sex, where obese was >95th; overweight was 85th to the 94th; healthy weight was 5th to the 84th; underweight was <5th (20).

### Acanthosis nigricans

Acanthosis nigricans (AN) was measured on the back of the neck by the Burke method on a 0–4 scale and analyzed as present (1–4) or absent (0) (21).

### Dietary intake

Dietary records were obtained on a subsample of 3520 children from the 11 jurisdictions. Two days of food records were completed by the caregiver, with assistance from other child caregivers as previously described (22). Caregivers were provided with measurement tools (cups and spoons, a ruler on the recording booklet, and a sealable plastic bag for collecting wrappers, labels, and packages). The booklet contained an example entry. Wrappers, labels, and packages of foods were collected during the CHL Program and used to aid in entry of the food records (22).

These data were entered into the Pacific Tracker version 3 (PacTrac3) application (23, 24) totaling 210,395 food items recorded on 13,673 days of food records for the CHL Program. PacTrac uses the food composition database that was developed and is maintained by the Nutrition Support Shared Resource at the University of Hawai‘i Cancer Center, which includes information from the USDA as well as on local foods identified by our group from children in the Pacific region (25, 26). Food composition data from foods identified in our studies—predominantly though not exclusively in children—have been added from published food composition data from other laboratories that follow national and international guidelines for food composition analysis and database development (9, 27). Nutrient intakes and food groups of the children's diet were calculated from 2 days, on randomly assigned days, to ensure representation of all days of the week across children. The dietary components were averaged across days, weighted for weekday and weekend days, and adjusted for within-person variance (23).

Macronutrient nutrient intakes (carbohydrate with starch as a subcategory, protein, and fat with saturated fat, monounsaturated fat, polyunsaturated fat, omega-3 (ω-3; n–3) fatty acids, ω-6 fatty acids as subcategories) were evaluated against DRI values (28). Food groups [grains, vegetables (including starchy vegetables), fruits, milk, and meats] were determined according to MyPlate food components of the Healthy Eating Index 2005 (29) and evaluated against Dietary Guidelines for Americans (30). The MyPyramid Equivalents Database includes 32/100 g equivalent measures for 7752 different foods, reported in ounce (oz) and cup equivalents accordingly (31).

The top 5 most frequently consumed foods were selected by food group based on the number of children that reported a specific food item in the dietary records. Foods were taken from PacTrac food descriptions, which were classified into a food class (e.g. white rice, potatoes, other tubers, banana, other fruit juices, canned fish, fresh fish), and then more broadly categorized into a food group (i.e. grain, vegetable, fruit, milk/dairy, or meats/animal products) using a classification method developed by University of Hawai‘i nutritionists. Vegetables included starchy vegetables and tubers (e.g., potatoes, taro, cassava, breadfruit), and condiments (e.g., shoyu or soy sauce, tomato ketchup). Fruits included fruit juices and coconut products (e.g., coconut meat, coconut milk). Milk/dairy included all dairy products. Meats/animal products included red meat, poultry, fish, eggs, and condiments made with eggs (e.g., mayonnaise). Mixed dishes, such as macaroni and cheese or pepperoni pizza, were not included in this analysis of frequently consumed foods.

### Statistical procedures

Prevalence and means were estimated using survey sampling techniques that weighted the sample to the young child population size in each community, based on census data, and accounted for the clustering of participants in communities within jurisdictions. The weighting resulted in representative estimates for the jurisdictions and the region, with 2 caveats. In Hawai‘i, the communities represented rural communities, and in Alaska the communities represented the most populous areas to meet the Intervention Study sampling criteria. Hierarchical marginal logistic models, adjusting for age and sex, of prevalence meeting recommendations were fit to compare subgroups and used a Wald test to evaluate the statistical significance for the interaction terms. Hierarchical marginal linear models, adjusting for age, sex, and dietary energy, of dietary components were fit to compare means between subgroups. Distributions of dietary variables were checked to ensure that model assumptions were met; none required transformation. Dietary variables were compared between jurisdictions, WBIGs, overweight and obesity (OWOB) compared with healthy-weight children, and children with AN compared with those without AN, to determine their dietary status. Underweight children were excluded from the analysis of OWOB compared with healthy-weight children. A *P* value <0.05 was considered statistically significant. Statistical analysis was done using SAS 9.4 (SAS Institute Inc).

Ethical approval was obtained from the Committee on Human Studies at the University of Hawai‘i at Manoa and the Institutional Review Board (IRB) at the University of Guam. All other participating jurisdictions ceded IRB approval to the University of Hawai‘i at Manoa. Parents consented and children assented to participate. Participants received $20 for participation in most locations. In Guam and Alaska, remuneration was increased to $40 and $50, respectively.

## Results

### Study population

The eligible study population of children aged 2–8 y included 3520 children (Table 1) of approximately equal numbers of males and females; 62% were 2–5 y old. The sample was predominantly indigenous Pacific Islander (Table 1), that is, indigenous to the jurisdiction where data were collected (16). Sixty-one percent of the households in the sample earned <US$35,000 per year, reflecting the lower income predominance of the sample.

The overall percentage with underweight in the LMI jurisdictions was 4%, in UMI was 2%, and in HI was 3%. The overall percentage with overweight in LMI was 10%, in UMI was 13%, and in HI was 14%. The overall percentage with obesity in LMI was 4%, in UMI was 19%, and in HI was 14%. The overall percentage with AN in LMI was 6%, in UMI was 9%, and in HI was 5%.

### Food groups

**Table 2** displays macronutrient and food group intakes by jurisdictions and by WBIG. Grain and meat food group intakes were higher in LMI jurisdictions whereas fruit and milk food group intakes were higher in HI jurisdictions. Children in Yap had a high consumption of meat (9.33 oz/d). Children in Chuuk had a high consumption of grains (8.22 oz/d), but very low consumption of milk (0.09 cups/d). Fruit intake was high in Alaska and American Samoa (1.28 and 1.26 cups/d, respectively). Overall, vegetable intake was low in all jurisdictions, <1 cup/d in each. Children in Alaska and American Samoa had the highest vegetable consumption of the region. Intake of starch (as a nutrient) was higher in LMI and lower in HI jurisdictions compared with UMI jurisdictions ( *P* < 0.005 based on the t-statistic).

**TABLE 2:** Adjusted child dietary intakes by Pacific jurisdiction and World Bank income groups^1^

Dietary intake	Lower-middle-income jurisdictions				Upper-middle-income jurisdictions			High-income jurisdictions				Total
	Chuuk	Kosrae	Pohnpei	Yap	American Samoa	Palau	RMI	Alaska	CNMI	Guam	Hawai‘i	
Total, *n*	123	125	187	190	588	166	191	308	546	666	430	3520
Food group daily intake^2^												
Grains, oz	8.22 (0.01)	7.18 (0.01)	7.90 (0.01)	7.19 (0.00)	7.64 (0.21)	6.50 (0.01)	7.24 (0.02)	5.88 (0.04)	6.71 (0.13)	6.57 (0.12)	6.28 (0.19)	6.93 (0.06)
Vegetables, cup	0.39 (0.00)	0.36 (0.00)	0.44 (0.00)	0.73 (0.00)	0.80 (0.03)	0.53 (0.00)	0.22 (0.00)	0.78 (0.02)	0.62 (0.04)	0.57 (0.02)	0.72 (0.01)	0.61 (0.01)
Fruits, cup	0.25 (0.00)	0.69 (0.00)	0.58 (0.00)	0.56 (0.00)	1.26 (0.04)	0.54 (0.00)	0.34 (0.00)	1.28 (0.04)	0.93 (0.02)	0.87 (0.03)	1.05 (0.06)	0.86 (0.02)
Milk, cup	0.09 (0.00)	0.58 (0.00)	0.51 (0.00)	0.58 (0.00)	1.48 (0.03)	0.91 (0.00)	0.55 (0.01)	1.70 (0.01)	1.09 (0.05)	1.23 (0.03)	1.48 (0.03)	1.07 (0.02)
Meats, oz	6.87 (0.01)	6.84 (0.01)	7.42 (0.01)	9.33 (0.00)	7.56 (0.31)	6.62 (0.01)	6.79 (0.02)	4.79 (0.06)	6.35 (0.21)	5.60 (0.08)	5.11 (0.14)	6.50 (0.09)
Percentage of energy from macronutrients per day^1^												
Carbohydrates (% energy)	59.65 (0.03)	54.86 (0.02)	54.78 (0.02)	52.86 (0.01)	53.59 (0.28)	51.71 (0.02)	52.03 (0.04)	51.97 (1.05)	51.67 (0.27)	52.52 (0.73)	53.33 (0.42)	53.1 (0.16)
Starch (% energy)	44.21 (0.04)	34.40 (0.03)	37.74 (0.02)	35.67 (0.01)	28.06 (0.19)	31.29 (0.02)	35.19 (0.04)	23.30 (0.04)	30.93 (0.18)	29.03 (0.34)	26.27 (0.34)	30.90 (0.21)
Protein (% energy)	14.83 (0.01)	15.20 (0.01)	16.29 (0.01)	16.25 (0.00)	14.82 (0.30)	16.43 (0.01)	15.56 (0.02)	15.96 (0.20)	15.82 (0.12)	15.10 (0.23)	15.27 (0.10)	15.52 (0.05)
Total fat (% energy)	25.04 (0.02)	30.26 (0.02)	28.48 (0.01)	30.01 (0.01)	31.71 (0.13)	31.85 (0.01)	31.69 (0.03)	33.38 (0.57)	32.51 (0.24)	32.77 (0.38)	32.42 (0.28)	31.54 (0.13)
Omega-3 fatty acids (% energy)	0.64 (0.00)	0.67 (0.00)	0.64 (0.00)	0.65 (0.00)	0.63 (0.01)	0.64 (0.00)	0.61 (0.00)	0.62 (0.02)	0.63 (0.01)	0.62 (0.01)	0.63 (0.01)	0.63 (0.00)
Omega-6 fatty acids (% energy)	4.16 (0.00)	5.45 (0.00)	4.59 (0.00)	4.77 (0.00)	5.68 (0.05)	5.43 (0.00)	5.20 (0.01)	5.64 (0.08)	5.60 (0.09)	5.70 (0.09)	5.61 (0.05)	5.40 (0.03)
Monounsaturated fat (% energy)	8.82 (0.01)	11.36 (0.01)	10.73 (0.01)	11.40 (0.00)	11.27 (0.06)	12.31 (0.01)	12.26 (0.02)	12.26 (0.20)	12.59 (0.19)	12.48 (0.14)	12.06 (0.13)	11.84 (0.06)
Polyunsaturated fat (% energy)	4.70 (0.00)	6.05 (0.00)	5.17 (0.00)	5.38 (0.00)	6.30 (0.05)	5.98 (0.00)	5.71 (0.01)	6.22 (0.10)	6.18 (0.12)	6.25 (0.10)	6.18 (0.06)	5.98 (0.04)
Saturated fat (% energy)	8.84 (0.01)	10.42 (0.01)	10.24 (0.01)	10.78 (0.00)	11.13 (0.14)	11.04 (0.01)	11.26 (0.02)	12.13 (0.20)	11.03 (0.07)	11.34 (0.15)	11.44 (0.20)	11.05 (0.05)

1 Values are means (SE). CNMI, Commonwealth of the Northern Mariana Islands; RMI, Republic of the Marshall Islands.

2 Adjusted for age and sex.

**FIGURE 2:**
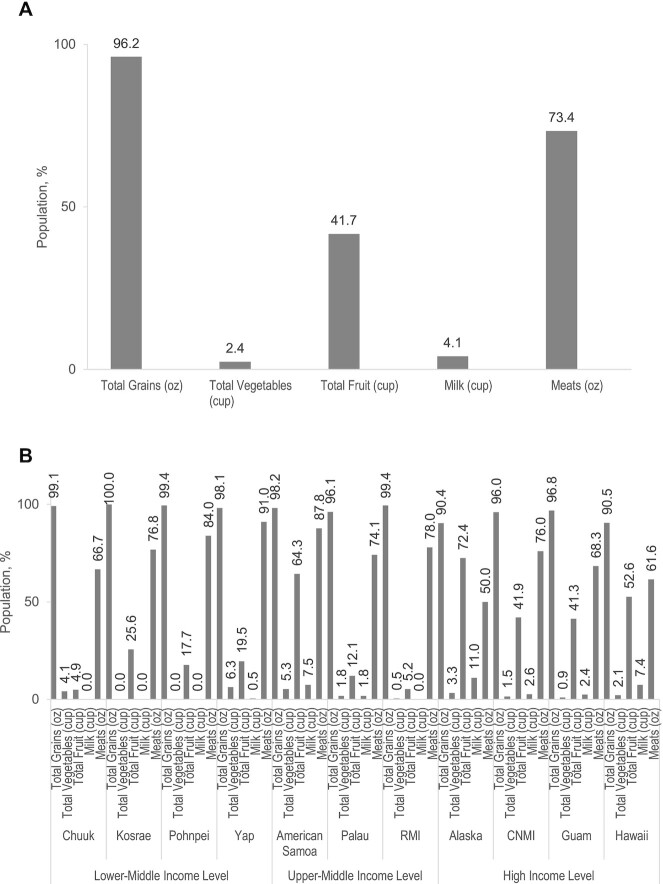
Food group intake above recommendations (41) in the (A) full sample (*n* = 3520) and (B) by jurisdiction in the US-Affiliated Pacific, Children's Healthy Living Program. All food groups, except meat, were significantly different between jurisdictions and World Bank income group (*P* < 0.05, Wald test). Jurisdiction sample: Chuuk (*n* = 123), Kosrae (*n* = 125), Pohnpei (*n* = 187), Yap (*n* = 190), American Samoa (*n* = 588), Palau (*n* = 166), Marshall Islands (*n* = 191), Alaska (*n* = 308), CNMI (*n* = 546), Guam (*n* = 666), and Hawai‘i (*n* = 430). CNMI, Commonwealth of the Northern Mariana Islands; RMI, Republic of the Marshall Islands.

**Figure 2** shows that whereas most of the population met food group intake recommendations for meats (73.4%) and grains (96.2%), very fewer children met food group recommendations for vegetables (2.4%), fruit (41.7%), or milk (4.1%) (*P* < 0.05).

White rice was the most frequently consumed type of grain food in all jurisdictions except Alaska, where white bread was most frequently consumed (**Table 3**). Reduced-fat 2% milk was the most frequently consumed type of milk in all jurisdictions except RMI and Chuuk, where whole milk and ice cream, respectively, were the most frequently consumed. Overall, the most frequently consumed fruit was bananas. In Palau, Guam, and Alaska children most frequently ate apples whereas in RMI and Chuuk, coconut was the most frequently consumed fruit. Other miscellaneous vegetables, which include cucumbers and mixed vegetables, were the most frequently consumed vegetable overall and most frequently consumed in Pohnpei and Hawai‘i. In HI jurisdictions, soy sauce (in CNMI and Guam) or potatoes (in Alaska) were the most frequently consumed vegetable, whereas in LMI jurisdictions, other tubers (in Chuuk, Kosrae, and Yap) were the most frequently consumed vegetables. Onions were the most frequently consumed vegetable in UMI jurisdictions. The type of meats consumed varied among jurisdictions. Chicken was the most frequently consumed meat among all jurisdictions overall and in all HI jurisdictions and most UMI jurisdictions (American Samoa and RMI). Canned fish was the most frequently consumed meat type in LMI jurisdictions of Chuuk and Yap. Fresh fish was the most frequently consumed meat in Pohnpei and Palau.

**TABLE 3:** Top 5 foods most frequently consumed foods among children by Pacific jurisdiction and World Bank income groups^1^

Lower-middle-income jurisdictions				Upper-middle-income jurisdictions			High-income jurisdictions				Overall
Chuuk	Kosrae	Pohnpei	Yap	American Samoa	Palau	RMI	Alaska	CNMI	Guam	Hawai‘i	
Food (*n*)	Food (*n*)	Food (*n*)	Food (*n*)	Food (*n*)	Food (*n*)	Food (*n*)	Food (*n*)	Food (*n*)	Food (*n*)	Food (*n*)	Food (*n*)
*Grains*											
1. White rice (124)	1. White rice (124)	1. White rice (205)	1. White rice (192)	1. White rice (560)	1. White rice (168)	1. White rice (189)	1. White bread (235)	1. White rice (543)	1. White rice (662)	1. White rice (362)	1. White rice (3201)
2. White bread (38)	2. White bread (59)	2. Other bread^2^ (95)	2. White bread (40)	2. White bread (435)	2. White bread (93)	2. Pancakes, waffles, or French toast (95)	2. Other cereal^3^ (212)	2. White bread (284)	2. Other cereal^3^ (403)	2. Other cereal^3^ (258)	2. White bread (1932)
3. Other bread^2^ (31)	3. Pancakes, waffles, or French toast (34)	3. White bread (56)	3. Other bread^2^ (29)	3. Other cereal^3^ (321)	3. Other cereal^3^ (71)	3. Other bread^2^ (87)	3. Crackers (166)	3. Other cereal^3^ (265)	3. White bread (380)	3. White bread (255)	3. Other cereal^3^ (1618)
4. Crackers (25)	4. Other cereal^3^ (30)	4. Crackers (31)	4. Other cereal^3^ (19)	4. Other bread^2^ (232)	4. Other bread^2^ (30)	4. White bread (57)	4. Pancakes, waffles, or French toast (107)	4. Crackers (99)	4. Other bread^2^ (143)	4. Crackers (172)	4. Other bread^2^ (974)
5. Pasta^4^ (19)	5. Other bread^2^ (27)	5. Other cereal^3^ (19)	5. Crackers (17)	5. Pancakes, waffles, or French toast (203)	5. Pancakes, waffles, or French toast (28)	5. Other cereal^3^ (20)	5. Whole wheat bread (103)	5. Other bread^2^ (91)	5. Crackers (128)	5. Other bread^2^ (117)	5. Crackers (824)
*Vegetables*											
1. Other tubers^5^ (48)	1. Other tubers^5^ (56)	1. Onion (61)	1. Other tubers^5^ (116)	1. Onion (202)	1. Onion (38)	1. Onion (28)	1. Potatoes (145)	1. Soy sauce (163)	1. Soy sauce (203)	1. Other miscellaneous vegetables^6^ (143)	1. Other miscellaneous vegetables^6^ (892)
2. Soy sauce (21)	2. Other miscellaneous vegetables^6^ (29)	2. Other miscellaneous vegetables^6^ (58)	2. Other dark-green vegetables^7^ (75)	2. Other tubers^5^ (188)	2. Light-green cruciferous (34)	2. Other tubers^5^ (21)	2. Carrots (130)	2. Other miscellaneous vegetables^6^ (145)	2. Potatoes (157)	2. Light-green lettuce (142)	2. Potatoes (702)
3. Onion (17)	3. Onion (13)	3. Other tubers^5^ (52)	3. Other miscellaneous vegetables^6^; soy sauce (71 each)^8^	3. Other miscellaneous vegetables^6^ (186)	3. Soy sauce (33)	3. Soy sauce (16)	3. Other light-green vegetables^9^ (120)	3. Potatoes (117)	3. Other miscellaneous vegetables^6^ (133)	3. Potatoes (130)	3. Soy sauce (684)
4. Spinach (15)	4. Light-green cruciferous (10)	4. Soy sauce (41)	4. Onion (45)	4. Potatoes (128)	4. Other miscellaneous vegetables^6^ (32)	4. Other light-green vegetables^9^ (10)	4. Light-green lettuce (85)	4. Carrots (90)	4. Tomatoes (106)	4. Tomatoes (128)	4. Onion (625)
5. Other miscellaneous vegetables^6^ (13)	5. Tomatoes (4)	5. Other dark-green vegetables^7^ (17)	5. Other light-green vegetables^9^ (35)	5. Corn (123)	5. Other tubers^5^ (27)	5. Pumpkin (9)	5. Other miscellaneous vegetables^6^ (75)	5. Onion (89)	5. Light-green lettuce (103)	5. Carrots (100)	5. Other tubers^5^ (578)
*Fruits*											
1. Coconut (37)	1. Banana (48)	1. Other miscellaneous fruits^10^ (60)	1. Other fruit juice^11^ (81)	1. Banana (299)	1. Apples (42)	1. Coconut (35)	1. Apples (205)	1. Other fruit juice^11^ (175)	1. Apples (198)	1. Other miscellaneous fruits^10^ (178)	1. Banana (1102)
2. Other fruit juice^11^ (18)	2. Coconut (26)	2. Coconut (58)	2. Banana (55)	2. Apples (263)	2. Oranges; other miscellaneous fruits^10^ (28 each)^8^	2. Other fruit juice^11^ (29)	2. Other miscellaneous fruits^10^ (166)	2. Apples (151)	2. Banana (176)	2. Apples (170)	2. Apples (1067)
3. Banana (12)	3. Other fruit juice^11^ (17)	3. Banana (52)	3. Other miscellaneous fruits^10^ (52)	3. Other miscellaneous fruits^10^ (212)	3. Banana; other pink fruits^12^ (16 each)^8^	3. Other miscellaneous fruits^10^ (21)	3. Banana (128)	3. Other miscellaneous fruits^10^ (149)	3. Other miscellaneous fruits^10^ (172)	3. Banana (154)	3. Other miscellaneous fruits^10^ (1062)
4. Other miscellaneous fruits^10^ (10)	4. Mango; other miscellaneous fruits^10^ (14 each)^8^	4. Peaches (34)	4. Coconut (40)	4. Coconut (202)	4. Other fruit juice^11^ (13)	4. Banana (17)	4. Other fruit juice^11^ (108)	4. Banana (145)	4. Oranges (155)	4. Other fruit juice^11^ (136)	4. Other fruit juice^11^ (852)
5. Orange juice (4)	5. Oranges (11)	5. Mango (19)	5. Pineapple (18)	5. Yellow papaya (160)	5. Orange juice; peaches (11 each)^8^	5. Orange juice (10)	5. Oranges (89)	5. Oranges (122)	5. Other fruit juice^11^ (141)	5. Oranges (121)	5. Oranges (709)
*Milk/Dairy*											
1. Ice cream (8)	1. 2% Milk (44)	1. 2% Milk (65)	1. 2% Milk (77)	1. 2% Milk (332)	1. 2% Milk (103)	1. Whole milk (40)	1. 2% Milk (208)	1. 2% Milk (274)	1. 2% Milk (403)	1. 2% Milk (258)	1. 2% Milk (1793)
2. Cream (4)	2. Whole milk (29)	2. Whole milk (58)	2. Whole milk (32)	2. Low-fat milk (299)	2. Whole milk (34)	2. 2% Milk (27)	2. Low-fat milk (140)	2. Low-fat milk (214)	2. Low-fat milk (217)	2. Low-fat milk (161)	2. Low-fat milk (1105)
3. 2% Milk; whole milk (2 each)^8^	3. Ice milk (18)	3. Low-fat milk (36)	3. Ice cream (20)	3. Whole milk (174)	3. Ice cream (30)	3. Ice cream (20)	3. Other cheese^13^ (139)	3. Processed cheese (104)	3. Whole milk (99)	3. Yogurt (105)	3. Whole milk (635)
4. Ice milk; yogurt (1 each)^8^	4. Ice cream (11)	4. Ice cream (10)	4. Low-fat milk (6)	4. Ice cream (167)	4. Low-fat milk (25)	4. Low-fat milk (4)	4. Processed cheese (97)	4. Ice cream (70)	4. Processed cheese (96)	4. Ice cream (102)	4. Ice cream (582)
5. None	5. Low-fat milk (3)	5. Dairy sauces and soups (3)	5. Processed cheese (4)	5. Processed cheese (55)	5. Processed cheese (19)	5. Ice milk (3)	5. Yogurt (96)	5. Whole milk (57)	5. Ice cream (93)	5. Processed cheese (99)	5. Processed cheese (476)
*Meats*/Animal Products											
1. Canned fish (78)	1. Chicken (85)	1. Fresh fish (147)	1. Canned fish (126)	1. Chicken (409)	1. Fresh fish (99)	1. Chicken; sausage (109 each)^8^	1. Chicken (141)	1. Chicken (319)	1. Chicken (450)	1. Chicken (231)	1. Chicken (2044)
2. Fresh fish (73)	2. Sausage (57)	2. Canned fish (118)	2. Fresh fish (118)	2. Eggs (357)	2. Chicken (96)	2. Fresh fish (100)	2. Sausage (123)	2. Eggs (284)	2. Sausage (372)	2. Sausage (199)	2. Sausage (1684)
3. Chicken (42)	3. Canned fish (48)	3. Chicken (99)	3. Sausage (98)	3. Sausage (303)	3. Sausage (72)	3. Canned fish (97)	3. Beef (104)	3. Sausage (277)	3. Eggs (193)	3. Beef (158)	3. Eggs (1318)
4. Sausage (26)	4. Fresh fish (46)	4. Pork (61)	4. Luncheon meats (66)	4. Canned fish (218)	4. Eggs (64)	4. Eggs (76)	4. Eggs (99)	4. Pork (195)	4. Beef (189)	4. Eggs (138)	4. Fresh fish (1109)
5. Luncheon meats (32)	5. Eggs (27)	5. Luncheon meats (56)	5. Chicken (63)	5. Mayonnaise (189)	5. Canned fish (54)	5. Cured meat (ham, corned beef) (33)	5. Cured meat (ham, corned beef) (56)	5. Luncheon meats (163)	5. Cured meat (ham, corned beef) (175)	5. Luncheon meats (79)	5. Canned fish (905)

1 Most frequently consumed foods presented as number of children (*n*) that reported consuming a food. CNMI, Commonwealth of the Northern Mariana Islands; RMI, Republic of the Marshall Islands..

2 Other bread food items may include doughnuts, biscuits, cinnamon rolls, and/or sweet bread..

3 Other cereal food items may include corn flakes, Cheerios, Froot Loops, Rice Krispies, and/or Honey Bunches of Oats.

4 Pasta food items may include spaghetti, macaroni, ramen, saimin, and/or soba.

5 Other tubers food items may include taro, breadfruit, cassava, plantains, and/or yams.

6 Other miscellaneous vegetables food items may include cucumber, mixed vegetables, eggplant, and/or mushrooms.

7 Other dark-green vegetables food items may include water spinach, kan kong, hibiscus leaves, and/or sweet potato leaves.

8 More than one food item is listed if the frequency of food items reported (*n*) is the same number.

9 Other light-green vegetables food items may include green beans, green peas, celery, string bean, and/or okra.

10 Other miscellaneous fruits food items may include grapes, green banana, honeydew melon, fruit salad, fruit leather, and/or pandanus paste.

11 Other fruit juice food items may include apple juice, coconut water, grape juice, pineapple juice, and/or passion-orange-guava nectar.

12 Other pink fruits food items may include watermelon and/or guava.

13 Other cheese food items may include cheddar, parmesan, provolone, Colby, Monterey Jack, Swiss, and/or cream cheese.

### Macronutrients

**FIGURE 3:**
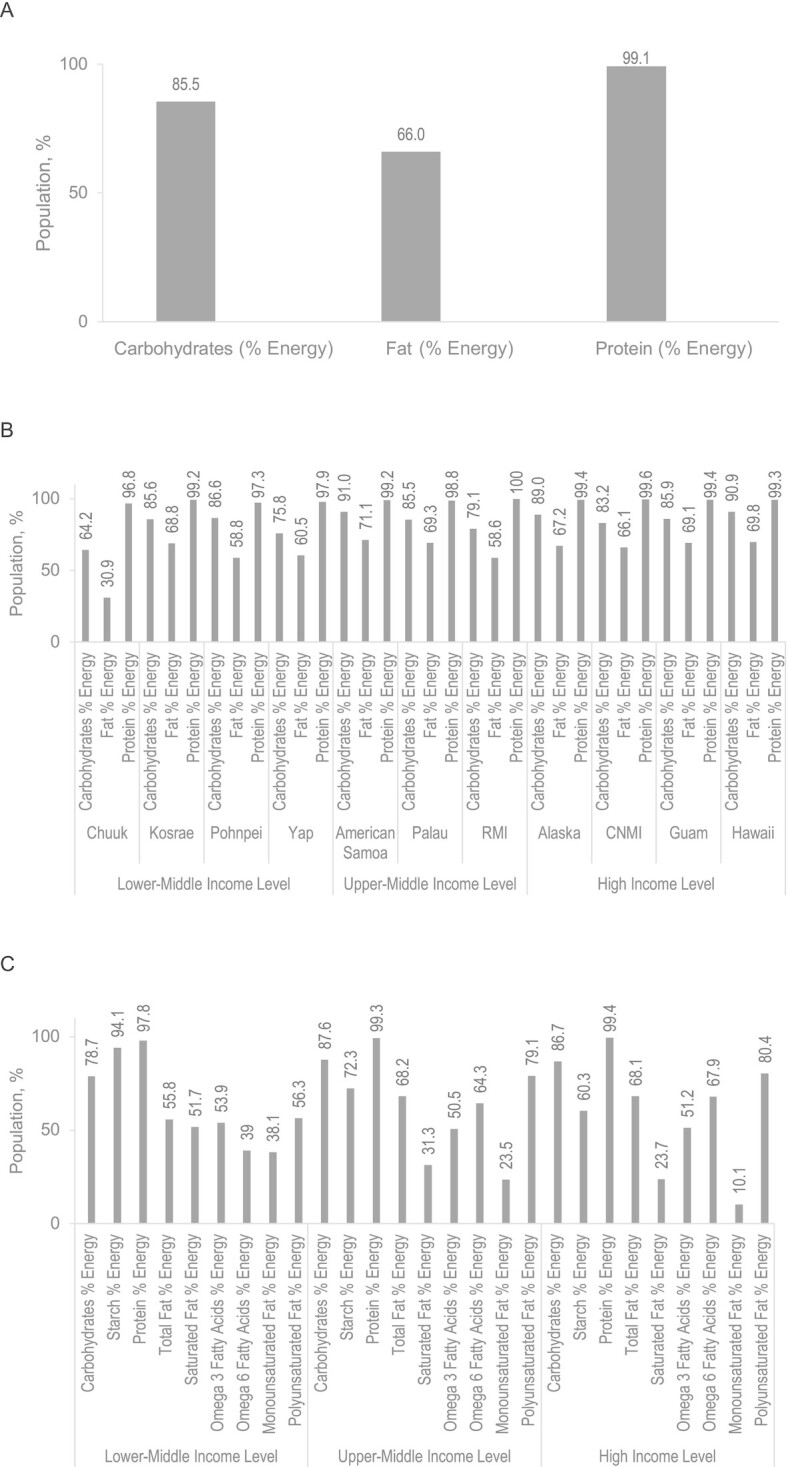
Macronutrient intake as a percentage of energy above recommendation (42) in (A) the full sample (*n* = 3520), (B) by Pacific jurisdiction and World Bank income groups, and (C) by macronutrient type in the Children's Healthy Living Program. In (B) carbohydrates and fats are significantly different between jurisdictions (*P* < 0.05, Wald test). In (C) all macronutrients shown, except protein, are significantly different between jurisdiction and World Bank income group (*P* < 0.05, Wald test). Jurisdiction sample: Chuuk (*n* = 123), Kosrae (*n* = 125), Pohnpei (*n* = 187), Yap (*n* = 190), American Samoa (*n* = 588), Palau (*n* = 166), Marshall Islands (*n* = 191), Alaska (*n* = 308), CNMI (*n* = 546), Guam (*n* = 666), and Hawai‘i (*n* = 430). CNMI, Commonwealth of the Northern Mariana Islands; RMI, Republic of the Marshall Islands.

**Figure 3** shows the percentage of children meeting recommended intakes for macronutrients. Consistent with food group intake findings, the greater part of the eligible study population was at or above recommended intakes for protein (99.1%), fat (66.0%), and carbohydrates (85.5%). Fat intakes varied by jurisdiction and WBIG, with an increasing percentage of energy from total fat with higher WBIGs. A total of 51.7% of children in the LMI jurisdictions met the dietary recommendations for saturated fat whereas 79.1% and 80.4% of UMI and HI jurisdictions, respectively, met recommendations for polyunsaturated fat. The main source of fat was sausage/frankfurter, which was also the main source of saturated fat and together with SPAM^®^ was the main source of monounsaturated fat; the main source of polyunsaturated fat was regular mayonnaise, across all jurisdictions.

Grain intake of 7.25 (SE: 0.08) oz was higher (*P* < 0.05) and milk intake of 0.90 (SE: 0.05) cups was lower (*P* < 0.05) in children with OWOB compared with those without OWOB [grains: 7.17 (SE: 0.07) cups; and milk: 0.96 (SE: 0.04) cups] (**Table 4**). After adjusting for age and sex, grain intake was higher (*P* < 0.05) in those with OWOB for most jurisdictions, except American Samoa and Hawai‘i, compared with children with OWOB in Alaska. Grain intake recommendations were met by between 90.4% (Alaska) and 100% (Kosrae). Monounsaturated fat intake of 11.68 (SE: 0.10) % energy was higher (*P* < 0.05) in those with OWOB, compared with the healthy-weight group [11.56 (SE: 0.08) % energy; *P* < 0.05]. Grain intakes were higher in UMI groups and lower in HI groups, compared with LMI groups. Milk intake was relatively low, with 0% meeting recommended intake in Chuuk, Pohnpei, Kosrae, and RMI. Milk intake was not related to OWOB status when adjusted by WBIG (*P* = 0.31). Milk intake of 0.85 (SE: 0.05) cups was lower (*P* = 0.02) in children with AN compared with children without AN [0.95 (SE: 0.04) cups].

**TABLE 4:** Food group and percentage of energy from macronutrients (carbohydrate, fat, protein) consumed per day by metabolic status [overweight and obesity (OWOB) (25) and presence of acanthosis nigricans (AN) (40) status] in the US-Affiliated Pacific, adjusted for age, sex, dietary energy, and jurisdiction

Food or macronutrient group	OWOB *n* = 923	No OWOB *n* = 2449	*P* value	Acanthosis *n* = 192	No acanthosis *n* = 3299	*P* value
	Mean (SE)	Mean (SE)		Mean (SE)	Mean (SE)	
*Food group*						
Grains, oz	7.25 (0.08)	7.17 (0.07)	0.0481	7.35 (0.13)	7.18 (0.07)	0.0829
Vegetables, cup	0.58 (0.03)	0.57 (0.03)	0.5358	0.54 (0.03)	0.57 (0.03)	0.1827
Fruits, cup	0.77 (0.04)	0.78 (0.04)	0.6722	0.77 (0.05)	0.78 (0.04)	0.9618
Milk, cup	0.90 (0.05)	0.96 (0.04)	<0.0001	0.85 (0.07)	0.96 (0.04)	0.0283
Meats, oz	6.83 (0.07)	6.80 (0.08)	0.5534	6.86 (0.19)	6.79 (0.08)	0.6434
*Macronutrient group*						
Carbohydrate (% energy)	53.48 (0.22)	53.57 (0.14)	0.7167	53.61 (0.55)	53.57 (0.12)	0.9472
Starch (% energy)	32.57 (0.11)	32.30 (0.08)	0.1196	32.90 (0.39)	32.31 (0.07)	0.1977
Protein (% energy)	15.43 (0.13)	15.54 (0.13)	0.1799	15.49 (0.20)	15.51 (0.12)	0.8780
Total fat (% energy)	31.07 (0.14)	30.86 (0.08)	0.1660	30.83 (0.39)	30.90 (0.07)	0.8482
Omega-3 fatty acids (% energy)	0.63 (0.01)	0.63 (0.01)	0.5110	0.63 (0.01)	0.63 (0.01)	0.6853
Omega-6 fatty acids (% energy)	5.26 (0.08)	5.25 (0.05)	0.7483	5.16 (0.09)	5.25 (0.05)	0.1940
Monounsaturated fat (% energy)	11.68 (0.10)	11.56 (0.08)	0.0475	11.73 (0.19)	11.58 (0.08)	0.3688
Polyunsaturated fat (% energy)	5.84 (0.08)	5.82 (0.05)	0.6899	5.73 (0.10)	5.83 (0.05)	0.1806
Saturated fat (% energy)	10.88 (0.14)	10.87 (0.09)	0.8800	10.79 (0.20)	10.88 (0.10)	0.5491

**TABLE 5:** Food group and percentage of energy from macronutrients (carbohydrate, fat, protein) consumed per day by metabolic status [overweight and obesity (OWOB) (25) and presence of acanthosis nigricans (AN) (40) status] in the US-Affiliated Pacific, adjusted for age, sex, dietary energy, and stratified by World Bank income group (all mean intakes differ by metabolic status and income group, Wald test, *P* < 0.05)

Food or macronutrient group	Lower-middle-income jurisdictions				Upper-middle-income jurisdictions				High-income jurisdictions			
	OWOB	No OWOB	AN	No AN	OWOB	No OWOB	AN	No AN	OWOB	No OWOB	AN	No AN
	*n* = 81	*n* = 504	*n* = 34	*n* = 579	*n* = 296	*n* = 622	*n* = 80	*n* = 860	*n* = 546	*n* = 1323	*n* = 78	*n* = 1861
	Mean (SE)	Mean (SE)	Mean (SE)	Mean (SE)	Mean (SE)	Mean (SE)	Mean (SE)	Mean (SE)	Mean (SE)	Mean (SE)	Mean (SE)	Mean (SE)
*Food group*												
Grains, oz	8.01 (0.09)	7.73 (0.09)	8.54 (0.10)	7.72 (0.09)	6.61 (0.11)	7.05 (0.11)	6.87 (0.29)	6.93 (0.08)	6.71 (0.11)	6.67 (0.10)	6.92 (0.16)	6.66 (0.10)
Vegetables, cup	0.56 (0.03)	0.51 (0.03)	0.48 (0.03)	0.51 (0.03)	0.66 (0.02)	0.53 (0.04)	0.51 (0.07)	0.57 (0.03)	0.69 (0.03)	0.67 (0.03)	0.62 (0.05)	0.68 (0.03)
Fruits, cup	0.54 (0.05)	0.55 (0.05)	0.47 (0.05)	0.56 (0.05)	0.95 (0.04)	0.76 (0.06)	0.87 (0.16)	0.81 (0.05)	1.05 (0.06)	1.02 (0.05)	0.97 (0.10)	1.03 (0.06)
Milk, cup	0.46 (0.05)	0.50 (0.05)	0.49 (0.05)	0.49 (0.05)	1.20 (0.08)	1.00 (0.06)	1.02 (0.14)	1.06 (0.06)	1.31 (0.05)	1.37 (0.04)	1.01 (0.08)	1.37 (0.05)
Meats, oz	7.81 (0.08)	7.92 (0.08)	7.14 (0.10)	7.94 (0.09)	6.58 (0.17)	6.87 (0.09)	6.96 (0.23)	6.77 (0.10)	5.82 (0.10)	5.84 (0.11)	6.48 (0.33)	5.79 (0.11)
*Macronutrient group*												
Carbohydrate (% energy)	55.87 (0.01)	55.05 (0.02)	56.23 (0.04)	55.11 (0.01)	52.39 (0.30)	52.90 (0.13)	51.85 (0.16)	52.85 (0.10)	52.49 (0.36)	52.38 (0.32)	52.40 (1.25)	52.43 (0.27)
Starch (% energy)	38.04 (0.01)	37.55 (0.02)	39.22 (0.05)	37.50 (0.02)	29.08 (0.11)	31.39 (0.28)	30.05 (1.17)	30.82 (0.15)	28.04 (0.24)	28.09 (0.29)	31.07 (0.61)	27.89 (0.27)
Protein (% energy)	15.78 (0.10)	15.65 (0.10)	15.59 (0.11)	15.67 (0.10)	15.43 (0.18)	15.59 (0.14)	15.84 (0.15)	15.51 (0.13)	15.34 (0.14)	15.41 (0.11)	15.32 (0.20)	15.38 (0.12)
Total fat (% energy)	28.11 (0.01)	28.75 (0.01)	27.69 (0.03)	28.72 (0.01)	32.59 (0.19)	31.48 (0.09)	32.32 (0.17)	31.74 (0.10)	32.68 (0.22)	32.61 (0.18)	32.36 (0.95)	32.64 (0.15)
Omega-3 fatty acids (% energy)	0.62 (0.01)	0.65 (0.01)	0.58 (0.01)	0.65 (0.01)	0.64 (0.01)	0.63 (0.01)	0.64 (0.01)	0.63 (0.01)	0.63 (0.01)	0.62 (0.01)	0.64 (0.02)	0.62 (0.01)
Omega-6 fatty acids (% energy)	4.65 (0.05)	4.77 (0.05)	4.45 (0.05)	4.76 (0.05)	5.75 (0.05)	5.44 (0.05)	5.60 (0.11)	5.51 (0.05)	5.62 (0.11)	5.62 (0.07)	5.45 (0.18)	5.63 (0.07)
Monounsaturated fat (% energy)	10.35 (0.09)	10.76 (0.08)	10.84 (0.09)	10.70 (0.08)	11.99 (0.14)	11.72 (0.08)	12.08 (0.16)	11.77 (0.09)	12.37 (0.12)	12.32 (0.12)	12.51 (0.46)	12.32 (0.10)
Polyunsaturated fat (% energy)	5.20 (0.05)	5.37 (0.05)	4.89 (0.06)	5.37 (0.05)	6.34 (0.05)	6.00 (0.05)	6.18 (0.12)	6.09 (0.05)	6.21 (0.11)	6.19 (0.07)	6.08 (0.19)	6.20 (0.08)
Saturated fat (% energy)	10.05 (0.12)	10.18 (0.12)	9.82 (0.12)	10.19 (0.11)	11.32 (0.17)	11.07 (0.09)	11.24 (0.15)	11.13 (0.11)	11.34 (0.16)	11.38 (0.13)	11.06 (0.39)	11.39 (0.12)

Among macronutrients, OWOB was related to lower intake of monounsaturated fats. Compared with Alaska, lower intakes of monounsaturated fats were found in Yap, Chuuk, Pohnpei, Kosrae, American Samoa, and Hawai‘i. Examining WBIG instead of jurisdiction, a similar pattern was found, with LMI having lower intakes of monounsaturated fats and HI having higher intakes (**Table 5**).

## Discussion

The diets of children in the Pacific generally meet macronutrient requirements, although dietary patterns vary by jurisdiction, WBIG, and metabolic status, and food group examination reveals areas that could be improved. Overall, there was a higher fat intake in HI jurisdictions, although a larger component of that fat was from polyunsaturated fats, whereas saturated fat and monounsaturated fat intakes were higher in LMI jurisdictions. This finding is consistent with a study showing that HI Pacific countries have higher sales of edible oils (34). A World Health Organization report on Pacific food supply showed increases in availability of animal fats, which are saturated, and vegetable oils, which may vary in type (5). Changes in food supply are likely influencing dietary patterns in the USAP, consistent with findings of nutrition transition in other parts of the world (33).

The components of dietary intake varying by WBIG levels of the jurisdictions studied are also an illustration of nutrition transition (7, 33). This transition refers to shifts in dietary patterns that have been influenced by changes in food sources, modes of processing and distribution, physical activity, and socioeconomic status (7). Indeed, the differences in income level among these jurisdictions may have influenced the food systems and food environment differently, and this study suggests these differences are related to food and macronutrient intakes of children. More studies are needed to investigate how variation in Pacific food environments may affect dietary intake in jurisdictions by WBIG.

The jurisdictions with the most underweight (6–7%) were LMI and UMI, whereas the jurisdictions with the most overweight (16–18%) and obesity (15–25%) were UMI and HI. The jurisdictions with the highest AN prevalence (9–12%) were found across the WBIGs, although AN has been found strongly associated with OWOB (35). Acanthosis nigricans screening has been recommended for the assessment, treatment, and prevention of pediatric obesity, in addition to BMI, rather than insulin concentration, which fluctuates during growth (36).

Food group means, except meats, and macronutrient means, except protein, were higher in OWOB compared with healthy-weight groups. Milk intake was lower in those with OWOB compared with those without. Grain intake was higher in children with OWOB, adjusting for age, sex, food energy, and jurisdiction. Monounsaturated fat intake, characterized by sausage and frankfurters, was higher in those with OWOB.

White rice was the predominant grain consumed in children of this study sample. Shifting from white rice to the indigenous tubers (e.g., yam, sweet potato, taro), evident in the starch (nutrient) intake among the LMI jurisdictions, might provide a strategy to increase vegetable intake and improve nutrition. Provision of milk for the young school-age children, especially in place of high-fat meats, may also improve nutrition status. For example, a 2014 review of dairy product intake and health outcomes in children has shown a neutral or beneficial relation (37). Plausible mechanisms of action are through modified fat absorption, appetite, and/or metabolic activity of gut microbiota, and possibly substituting for sugar-sweetened beverages (37). Overall, shifting consumption from rice and meat to tubers and their leaves, and milk, might be a strategy to improve the nutritional status of USAP children. Tubers are locally grown vegetables, the production and consumption of which could be supported with local policies. Strategies for increasing milk consumption would likely require efforts on both production and consumption, and/or trade, aid, and/or importation, because production levels are low in the region. Shelf-stable milks may be a partial solution.

A strength of our study is the dietary assessment of an unstudied high-risk population, whereas dietary intake assessment of children that relies on caregiver reporting is a limitation.

Our findings show a need for improvement in young children's diets in the USAP, especially for intake of vegetables and milk. Because milk intake was lower in OWOB children, improving milk intake may help reduce prevalence of OWOB in children in the Pacific.

In conclusion, young children's diets in the USAP did not meet milk or vegetable intake recommendations. There was variability in dietary patterns across the USAP region by WBIG. Grain intake was higher and milk intake was lower in children with OWOB.

## Data Availability

Data described in the manuscript and data dictionary will be made available upon request pending approval by the CHL Program Steering Committee, agreement to data security requirements, and payment of processing fees.

## Notes

The Children's Healthy Living Program was funded by the Agriculture and Food Research Initiative Grant no. 2011-68001-30335 from the USDA National Institute of Food and Agricultural Science Enhancement, Coordinated Agricultural Program (PI: RN), and from the USDA National Institute of Food and Agriculture, Agriculture and Food Research Institute Grant no. 2018-69001-27551 (PI: RN).

Author disclosures: The authors report no conflicts of interest.

Abbreviations used: AN, acanthosis nigricans; CHL, Children's Healthy Living Program; CNMI, Commonwealth of the Northern Mariana Islands; FAS, Freely Associated States; GNIPC, gross national income per capita; HI, high-income; IRB, Institutional Review Board; LMI, lower-middle-income; NHOPI, Native Hawaiian and Other Pacific Islanders; OWOB, overweight and obesity; PacTrac3, Pacific Tracker version 3; PI, principal investigator; RMI, Republic of the Marshall Islands; UMI, upper-middle-income; USAP, US-Affiliated Pacific; WBIG, World Bank income group.
